# Acquiring Knowledge about the Use of a Newly Developed Electronic Fetal Heart Rate Monitor: A Qualitative Study Among Birth Attendants in Tanzania

**DOI:** 10.3390/ijerph15122863

**Published:** 2018-12-14

**Authors:** Sara Rivenes Lafontan, Johanne Sundby, Hussein L. Kidanto, Columba K. Mbekenga, Hege L. Ersdal

**Affiliations:** 1 Institute of Health and Society, Faculty of Medicine, University of Oslo, 0317 Oslo, Norway; Johanne.sundby@medisin.uio.no; 2 Medical College, East Africa, Aga Khan University, Dar es Salaam, P.O. Box 38129, Tanzania; hkidanto@gmail.com; 3 School of Nursing and Midwifery, Aga Khan University, Dar es Salaam, P.O. Box 38129, Tanzania; columba.mbekenga@aku.edu; 4 Department of Anesthesiology and Intensive Care, Stavanger University Hospital, 4011 Stavanger Norway; hege.ersdal@safer.net

**Keywords:** Tanzania, low-resource setting, labor care, (electronic) fetal heart rate monitoring, labor monitoring, health literacy, Moyo, wireless fetal heart rate monitor, birth attendant

## Abstract

In an effort to reduce newborn mortality, a newly developed strap-on electronic fetal heart rate monitor was introduced at several health facilities in Tanzania in 2015. Training sessions were organized to teach staff how to use the device in clinical settings. This study explores skilled birth attendants’ perceptions and experiences acquiring and transferring knowledge about the use of the monitor, also called Moyo. Knowledge about this learning process is crucial to further improve training programs and ensure correct, long-term use. Five Focus group discussions (FGDs) were carried out with doctors and nurse-midwives, who were using the monitor in the labor ward at two health facilities in Tanzania. The FGDs were analyzed using qualitative content analysis. The study revealed that the participants experienced the training about the device as useful but inadequate. Due to high turnover, a frequently mentioned challenge was that many of the birth attendants who were responsible for training others, were no longer working in the labor ward. Many participants expressed a need for refresher trainings, more practical exercises and more theory on labor management. The study highlights the need for frequent trainings sessions over time with focus on increasing overall knowledge in labor management to ensure correct use of the monitor over time.

## 1. Introduction

Recently, there has been an explosion in the development and implementation of mobile health solutions and technological devices to diagnose and treat diseases worldwide. Increasingly being used to tackle global health issues, innovation in health technologies is considered an important tool to achieve the health related United Nations Sustainable Development Goals in low- and middle-income countries [1,2]. Here, implementation of new technology is used to counter challenges in the health systems which impedes access to quality health services and by replacing outdated interventions that are slow and imprecise [3]. The development of an electronic strap-on fetal heart rate monitor, called Moyo (Appendix A) developed by Laerdal Global Health (Stavanger, Norway) is one such example. In an effort to reduce perinatal mortality, the monitor is developed for use in low resource settings, where two million babies die during labor (fresh stillbirths) and almost three million newborn babies die within their first month of life (neonatal deaths), [4]. Strapped on the abdomen of a woman in labor, it uses ultrasound technology to continuously measure fetal heart rate. It alerts the caregiver of fetal distress (i.e., slow heart rate or extremely high rate) by an alarm, to ensure timely interventions to prevent intra-uterine hypoxia leading to fresh stillbirths and birth asphyxia—one of the leading causes of newborn deaths [5,6]. Moyo was introduced in Tanzania in 2015, one of the countries in Sub-Saharan Africa with high newborn mortality rates [4].

A vital component in a successful adaptation of tools such as Moyo, is the complex process of internalizing knowledge about how to use and interpret the technological device. Knowledge about this process is crucial to further improve training programs and ensure long-term use, as many innovative technological solutions initially fail in this endeavor [7,8]. Through a review of literature, we have been unable to identify studies investigating skilled birth attendants’ perspectives of acquiring knowledge about the use of a technological device in low resource settings. However, a study from Tanzania about the use of the intermittent electronic fetal monitor, Doppler, found that midwives believed they had insufficient training to use it [9]. Hence, there is a need to better understand the process of learning to use new technology among skilled birth attendants. As part of an ongoing evaluation of the introduction of Moyo in Tanzania, the objective of this paper is to present skilled birth attendants perceptions and experiences acquiring knowledge about the use of the monitor and transferring this knowledge in the labor ward.

## 2. Materials and Methods

### 2.1. Study Design and Data Collection

The current study aimed to explore the perceptions and experiences of skilled birth attendants hence a qualitative approach was found to be most suitable. Focus group discussions (FGDs) was selected as data collection method as it allows a deeper understanding of social phenomena such as how people acquire knowledge, by obtaining the points of view of many individuals through a discussion initiated by the interviewer exploring experiences, views, motivations and beliefs on a specific topic [10].

In total, five FGDs were carried out with 4–6 participants in each group (Table 1). The FGDs took place at the hospital premises at both hospitals. An interview guide was used to guide the discussion and included open-ended questions starting with asking the participants how they had learnt to use Moyo, by whom and their motivations to learn about Moyo, probing for the approach used, time allocated and content. The participants were also asked if they had any recommendations for how the training could be improved. The discussions were conducted in Kiswahili by a research assistant who was a university teacher in midwifery with experience in conducting qualitative research. The first author was present during all interviews to observe and take notes. The FGDs lasted 60–80 min and took place at a location that ensured privacy at the two facilities. After each FGD, the facilitator and first author discussed responses to each question in order to make amendments to the interview guide if necessary. Data collection was carried out is until was believed that further data collection revealed no new themes and thematic saturation was reached [10]. The data collection took place from December 2016 to March 2017. The FGDs were audio recorded and transcribed verbatim and translated to English by an experienced transcriber and translator who were both trained by the first author. Transcripts and translated versions were verified by members of the research team.

### 2.2. Recruitment of Participants and Ethics

Skilled birth attendants working at the labor ward and who were using Moyo at the two study sites were invited to participate in the study. All those asked to participate accepted. In total, six medical doctors and 20 nurse midwives participated in the study. The medical doctors formed themselves into one FDG group.

The study was conducted following the ethical principles set out in the Declaration of Helsinki [11]. All participants received oral and written information about the purpose of the study before giving their written consent to participate. The Safer Births project has been granted ethical approval by the Norwegian Regional Ethics Committee (REK Vest; Ref: 2013/110/REK vest) and the Tanzanian National Institute for Medical Research (Ref: NIMR/HQ/R.8a/Vol.IX/388). The first author obtained a research permit to carry out the study from the Tanzania Commission for Science and Technology, COSTECH, (No. 2016-396-NA-2016-277). Permission to conduct the study was obtained from all relevant entities, both at the institutions where the study was carried out and the municipality. Permission to publish the final manuscript was obtained from the Tanzanian National Institute for Medical Research.

### 2.3. Study Setting

The study was conducted at two hospitals in Tanzania’s largest city, Dar es Salaam, a city with a population of 4.3 million. Hospital 1 is a tertiary referral hospital which receives patients referred from both public and private practice and also serves paying private patients. It has 10,000 annual deliveries. The obstetric department is staffed with 40 obstetric and gynecologic (Ob-Gyn) medical specialists who rotate on different wards, including the labor ward, resident doctors and intern doctors and 21 registered nurse-midwives and eight nurse attendants. Hospital 2 is a district referral hospital which receives referred patients from 135 surrounding health facilities within its catchment area of 2 million inhabitants. 25 nurse-midwives, five nurse attendants, eight registered doctors and two OB/GYN specialists are employed at the labor ward. There are about 17,000 deliveries taking place at this hospital every year.

### 2.4. Data Analysis

Data analysis was conducted in an iterative, inductive manner which started during the data collection. After re-reading the translated transcripts repeatedly in their entirety to deepen familiarity with the contents, a qualitative content analysis as described by Graneheim and Lundman [12] was applied to the material. Using the computer software package NVivo 11 (QSR International, Melbourne, Australia), the text was analyzed line by line and condensed into meaning units called codes. A list of codes was developed and the codes were compared to find patterns in the data. The codes were subsequently sorted into categories based on commonalities between codes. Efforts were made when developing codes and categories to maintain the “voice” of the participants. The first author (SRL) coded the data which was later shared and discussed among authors in an effort to reduce researcher bias. Table 2 provides an illustration of how codes, sub-categories and categories were created.

### 2.5. The Moyo Training

The Moyo training was initiated with two comprehensive training workshops organized by senior and management staff at the labor ward at the two hospitals. The first aimed at training master trainers, and the second included skilled birth attendants working in the labor ward at the two hospitals, organized in June and December 2015 respectively. Staff from the two hospitals were trained together in a separate location at one of the hospitals. During the initial Training of Trainers (ToT), the 20 master trainers were selected by senior staff at both maternity wards and were both nurse-midwives and medical doctors. These participants would then be responsible for training their colleagues at the two labor wards about the use of Moyo. The training curriculum included components of fetal heart rate monitoring, labor management and the functions of Moyo. More specifically, this included how to operate the device by correct placement of the probe on the abdomen, the different features of the device such the three buttons on the device namely the on/off button, history button showing the FHR during the past 30 min and the button to silent the alarm which rings during prolonged periods of abnormal fetal heart rate. Other issues included charging and cleaning of the device and instances when the device should not be used such as multi-fetal pregnancies. Flip-charts illustrating correct use of the device and Power Point presentations were used during the training. The workshop also included a demonstration at the labor ward showing how the device is strapped on a pregnant woman’s abdomen. The subsequent training of skilled birth attendants included much of the same curriculum as in the ToT, however a training report states that it was of shorter duration as participants by then had a basic understanding of how to use Moyo from the master trainers. In addition, shorter training sessions were carried out in-situ/in-house at the labor ward at the two hospitals during the course of 2016.

## 3. Results

### 3.1. Demographic Characteristics

The age range of participants was 22–48 years, average age was 37 years. Median years of experience in labor care was 4 years. Four participants were men and 22 women. A summary of participant characteristics is presented in Table 3 below.

### 3.2. Categories

Four main categories regarding the perceptions and experiences acquiring and transferring knowledge about the use of Moyo were identified. These were: (1) learning through different approaches; (2) colleagues motivation to learn; (3) the need for more training and (4) ways in which the Moyo training could be improved.

#### 3.2.1. Learning through Different Approaches

The participants in the study had acquired knowledge about how to use Moyo by attending either the ToT training, an in-house training session, through a colleague or by self-learning. Those who had attended the initial ToT training said the learning tools, such as flip-chart used for the training was reported as being easy to understand and therefore helpful in understanding how to operate the monitor. One issue which was frequently mentioned when discussing the initial ToT was that many of those who had participated in the ToT were no longer working in the labor wards. Many of the participants in the FGDs had not attended the ToT which they felt would have been beneficial. In one of the FGD groups, none of the participants reported having participated in the ToT training and reported knowing only 3 to 4 colleagues who had. At both study sites, it was often mentioned that more staff should have attended the ToT training to receive a more comprehensive training. According to a participant who had not attended the ToT training:

(The) *training was provided to very few people, so I would suggest that it is allocated to a lot more so that everyone is confident and sure about how to use Moyo.*

(Nurse-midwife, FGD 4)

Those who attended the in-house training said that while it included both a theoretical and a practical component practicing on a laboring woman, it was of shorter duration compared to the ToT training and it was frequently mentioned that it should have been longer. One doctor who attended the in-house training however, felt that the training was sufficient due to the small group, interactive teaching style of the facilitator, and availability of devices for each participant:

The mode of teaching was good because we were only a few people, and everyone had the device at hand while the facilitator was teaching, he also had a chart, everyone was able to participate and ask questions.

(Doctor, FGD 1)

#### 3.2.2. Colleagues Motivation to Learn

Those who had attended the ToT had to acquire knowledge about Moyo while also being responsible for training colleagues in the ward. Not everyone was interested in learning about Moyo and the participants explained how they evaluated the attitude of the colleagues and their motivation to be trained, before deciding how much details they would include in the training session. When asking one participant how much time she spent teaching each colleague she responded:

It depends on an individual’s awareness and willingness to learn.

(Nurse-Midwife, FGD 5)

One participant who had attended the ToT, believed that some colleagues were not interested in learning about Moyo and partly blamed the timing of the ToT training for this:

The ones who wants to learn will come to you and learn but you can’t force those who are not interested… The problem occurred in the beginning after a few people went to the (ToT) training. Those who didn’t attend lost interest and it was too late by the time they received training because they’d already lost interest by then.

(Nurse-Midwife, FGD 4)

#### 3.2.3. The Need for More Training

Some participants stated that they did not feel they had received enough training before starting to use Moyo with one participant stating:

I personally think there is a need to learn Moyo in detail, there are a lot of things such as differentiating maternal heart rate from fetal heart rate which can be easily mixed up…

(Nurse-Midwife, FGD 2)

Other participants mentioned that colleagues who did not know how to use the device, blamed the device when it did not function as they wanted it to:

They think that all you have to do is to place the device and it will read the fetal heart rate automatically, they are the ones who complain that Moyo doesn’t read properly.

(Nurse-Midwife, FDG 2)

Some of the participants seemed unaware of basic functions of Moyo. One doctor who had learnt about the monitor from a colleague was not aware of the fetal heart rate history function of the device, displayed by pressing one of the three buttons on the device. In one of the groups where several of the participants had attended a ToT training, there was a lengthy discussion about if the device can be used for twin pregnancies or not, an issue covered during the training.

Overall, the participants expressed a desire to spend more time on training. One aspect of this was to expand the duration of the Moyo training. However, it differed between participants if this time should be spent on more theory about labor management or more practice using Moyo. The participants also mentioned other areas of labor management where they would like to strengthen their knowledge. These included: how to detect the baby by pelvic examinations, how to ensure a safe delivery, the management of eclampsia and using ultrasound. One participant, who thought there had been enough theory during the in-house training, would like to learn more about the practical detection of abnormal fetal heart rate:

We need to spend more time with the patients and to learn all steps taken to detect fetal heart rate abnormalities.

(Nurse-Midwife, FGD 3)

#### 3.2.4. Suggestions for Ways in Which the Moyo Training Could Be Improved

When asked how the training about Moyo could be improved, several issues were mentioned. The most common feedback at both study sites was that there was a need for a refresher training. This was mentioned among participants who had attended both the in-house training and the initial ToT. Another common suggestion was that when practicing using Moyo, this should be done on pregnant women:

It is impossible to take in all the theoretical information, practice is necessary if someone wants to learn well.

(Nurse-Midwife, FDG 5)

One participant who had attended the ToT training said that there should have been more Moyos available during the training so each participant had one they could practice on. It was suggested that brochures, posters and instructional diagrams could be useful teaching aids in addition to the user guide that comes with the device. Two doctors would like to know more about the limitations of Moyo; when the device would provide false results and inaccurate readings.

## 4. Discussion

This study explored birth attendants’ perceptions and experiences acquiring and transferring knowledge about the use of a newly developed fetal heart rate monitor introduced at the labor ward. While the majority of the participants were positive towards the contents of the training they had received, a general perception was a need for additional training in order to become fully confident using the device. Several issues were mentioned regarding the ToT, such as staff turnover which resulted in few master trainers still remaining in the labor ward and lack of motivation to learn about the device among staff who had not been selected to attend the ToT. Among positive aspects mentioned was learning in smaller groups and being able to practice using the device on women in labor.

The participants’ desire to practice using Moyo on pregnant women can be interpreted as a wish to learn how to integrate the device into one’s current practice. To become an integral part of the way the birth attendants carry out their tasks, training modules about how to use a new technological device should incorporate not only how to operate the device but also practical exercises. We argue that the process of integrating Moyo into their practice requires the same complex learning process as taking up any other new procedure into one’s established, well learned and institutionalized practice. Changing behavioral practices takes time. In a study from Zanzibar where birth attendants were trained in locally adapted intrapartum guidelines, four-hour training sessions were conducted on a quarterly basis and were continued after the end of the study [13]. Bandura’s social cognitive theory of self-efficacy is often used to understand mechanisms of learning by focusing on personal factors, environmental factors and behavior [14]. Self-efficacy, according to Bandura, is the extent to which a person has confidence that it can perform a task set before him or her and is a prerequisite for learning. It is the result of a multifaceted process which includes previous experience, self-esteem, perception of self and phyco-social factors and overall health. Students who are motivated display greater progress than unmotivated students [15]. Additionally, slow learners are less likely to seek help because it might expose their limitations. One can hypothesize that birth attendants with low self-efficacy were reluctant to learn about Moyo, which might be interpreted by colleagues as a lack of motivation. The reported lack of motivation to learn about the device could also be linked to a lack of confidence in how to respond to sins of fetal distress and or labor management in general. Increasing knowledge in this area might therefore have been beneficial to fully benefit from the advantages of using the device and improve overall care. The fact that several of the participants revealed a desire to learn more about areas of labor management indicates that not all of the participants in the study feel confident in aspects of labor care required to perform their duties. Being considered easy to use, one can wonder if the expressed need to *learn Moyo more in detail* could also be a desire to increase knowledge about labor monitoring and how to respond to fetal distress in particular and not necessarily a need to learn more about how to operate the device. The process of acquiring knowledge about Moyo could have generated a need to strengthen knowledge in other areas of the participants clinical practice. However, with better knowledge about the status of the fetus, also comes a responsibility to carry out the correct follow-up actions. In low-resource settings, the lack of resources has been found to be an obstacle to implementation of new knowledge. This, combined with a fear of being blamed for negative fetal outcome, might impede the birth attendant from implementing the correct obstetric actions [16,17].

The participants in the study expressed a need for additional training which could be due to the time lapse of a year from the training sessions took place to the time when the FGDs were conducted. However, it is similar to findings from a study conducted among doctors in Rwanda about the use of ultrasound in labor care, which found that many wanted more training to improve ultrasound skills [18]. A review of retention of knowledge of newborn resuscitation and a review of simulation training in post-natal care also found that the participants thought the training should be longer [19,20]. One potential consequence of inadequate training from our results is the potential to blame user errors on the device with participants believing the device provided inaccurate or incorrect results. This is an area we believe should be studied further. It should also be of particular concern to those who introduce new technology, as a lack of understanding of how to use the device make successful adaptation unlikely.

The need for refresher trainings due to high turnover of staff has also been found in studies conducted among birth attendants in Tanzania after simulation training in neonatal resuscitation [9,21]. Evaluations of Helping Babies Breathe found that a one-day training was not sufficient to incorporate new skills into practice, which highlights the need for sustained opportunities to build skills and knowledge through training [22,23]. We therefore recommend using what has been called “low dose/high frequency” training when introducing new technological devices in the labor ward in similar settings. This model, which also relies on training of trainers, has been used for simulation training to improve newborn survival in Tanzania and elsewhere with positive results [20,23,24,25]. Conducting regular, shorter training sessions in a systematic manner would have allowed staff to regularly practice using Moyo, and also created a forum to discuss any issues experienced using the new device. This type of frequent, short training sessions could also help foster a learning culture and increase motivation to learn while preventing loss of institutional memory about how to use the device being lost due to turnover.

Our findings indicate it would have been beneficial to include a component of motivation and learning theory in the ToT module. This cascade training program where master trainers are given the responsibility to ensure that colleagues are trained, is a commonly used, cost-effective way of diffusing knowledge in organizations. While its usefulness has been documented [26], it does require effective supervisory and pedagogical skills by the master trainers. If these skills are lacking, the master trainers might be unable to effectively approach colleagues perceived to be less motivated. However, increasing birth attendants’ motivation to learn should not only be a priority during training sessions. Lack of overall motivation has been found among birth attendants in Tanzania and should be of concern [27]. One way to improve both practice and motivation conducive to learning, is through supportive supervision. A review paper from Sub- Saharan Africa on the effects of supportive supervision, found that it can increase job satisfaction and health worker motivation [15]. In addition, it was found that providers who received training on quality improvement tools such as new technological devices, were motivated compared to those who did not, which is similar to our findings [28]. Through supportive supervision, participants would also be able to receive feedback and discuss the way in which Moyo was used after attending training sessions. This could in turn facilitate a continued learning process where the device, and the correct follow-up actions to its alerts of fetal distress, became integrated into the birth attendants’ clinical practice.

### Strengths and Limitations

Strengths of the study include the use of FGD as a data collection method which was suitable, allowing for interaction between participants and gathering of different perspectives and views about the study objective. A broad range of study participants including both doctors and nurse/midwives, males and females, with a various degree of experience in labor care and from two health facilities at different levels of the healthcare system were included in the study to increase credibility of the study findings. Since the discussions were conducted in Kiswahili, the participants were able to express themselves with ease during the discussions in their native tongue. The study was conducted by a multi professional and multi- cultural team which brought both insider and outsider perspectives to the process and also increased credibility of the study findings. The fact that the first author, although not fluent in Kiswahili, attended and took notes during all FGDs strengthened the analytical process. Limitations of the study include the possible recall bias as some of the participants had undergone training one year before the study was carried out. Meanings might also have been lost during the process of transcribing and translating the FGDs, despite efforts made to counter this by training the transcriber and translator and verifying both transcripts and translations by other members of the research team.

## 5. Conclusions

The participants in this study expressed a need to strengthen their knowledge about the use of a new electronic fetal heart rate monitor, despite most participants having participated in organized training sessions about how to use the device. More time spent on training birth attendants about the use of the electronic fetal heart rate monitor would have been beneficial. Specifically shorter and more frequent training sessions is recommended to increase learning output. The participants in the study also expressed a need to obtain further training in other areas of labor management, which indicates an unmet need for knowledge in aspects of their practice which should be addressed to ensure a skilled and motivated workforce.

## Figures and Tables

**Table 1:** Data collection per study site.

	Data Collection	Medical Doctor	Nurse-Midwife
Study site 1	2 FDGs	0	9
Study site 2	3 FDGs	6	11

**Table 2:** Example of the analysis process.

Translated Transcribed Interview	Code	Category
*I personally think there is a need to learn Moyo in detail, there are a lot of things such as differentiating maternal heart rate from fetal heart rate which can be easily mixed up, but we could also use other devices like fetoscope to confirm.*	There is a need to learn more about Moyo	The need for more training *

Note: * the category was formed by several codes.

**Table 3:** Demographic description of participants by age group, gender and number of years of experience.

Characteristics		n (26)	%
Age	20–29	5	19
	30–40	8	31
	above 40	13	50
Gender	Female	22	85
	Male	4	15
Years of experience working in the labor ward	1	2	8
	2	3	12
	3	5	19
	4	7	27
	5	6	23
	above 5	3	11

**Figure A1:**
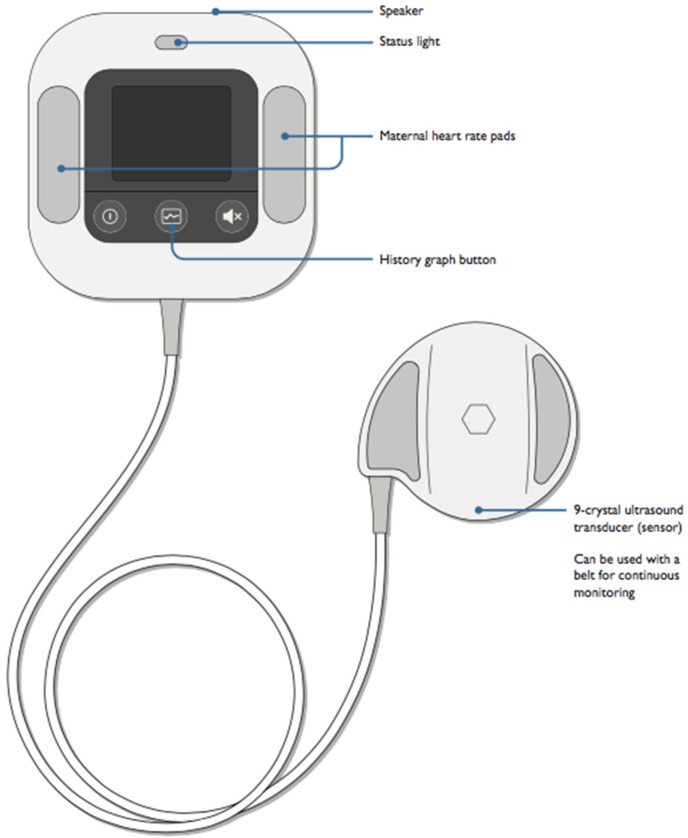
The Fetal Heart Rate (FHR) monitor, Moyo (Laerdal Global Health).
